# Epidemiologic Questions from Anthrax Outbreak, Hunter Valley, Australia

**DOI:** 10.3201/eid1505.081744

**Published:** 2009-05

**Authors:** David N. Durrheim, Paul Freeman, Ian Roth, Michael Hornitzky

**Affiliations:** University of Newcastle, Newcastle, New South Wales, Australia (D.N. Durrheim); New South Wales Department of Primary Industries, Wollongbar, New South Wales, Australia (P. Freeman); New South Wales Department of Primary Industries, Orange, New South Wales, Australia (I. Roth); New South Wales Department of Primary Industries, Camden, New South Wales, Australia (M. Hornitzky)

**Keywords:** Zoonoses, bacteria, anthrax, cattle reemergence, epidemiology, Australia, surveillance, letter

**To the Editor:** Anthrax was introduced into Australia in 1847 near Sydney, New South Wales, and spread along stock routes throughout New South Wales and southern Queensland (*1*). Anthrax was considered endemic to the Hunter Valley, New South Wales, during the 1890s. The last recorded anthrax-related stock losses there occurred on 3 properties in the Upper Hunter Valley in 1939 (*1*).

During the past 4 decades, anthrax has become uncommon in Australia. Clinical cases are seen only sporadically in sheep, cattle, and (rarely) horses. Annually, 6–12 properties are affected in unrelated incidents; where cattle are involved, generally only 1–3 animals per property are affected (*2*). Anthrax is confined almost exclusively to a belt running through the center of New South Wales (*3*).

From December 14, 2007, through January 3, 2008, a total of 53 cattle (*Bos taurus*) with peracute anthrax and 1 horse died on 11 properties in the Rouchel area, 20 km east of Aberdeen in the Hunter Valley and 350 km from the anthrax belt (Figure). The area is hilly, rising to ≈550 m, with alluvial soils alongside a stream and rocky basaltic and sandy soils on the slopes. Most properties feature gullies that flow intermittently after rain. The affected properties covered ≈60 km^2^.

**Figure:**
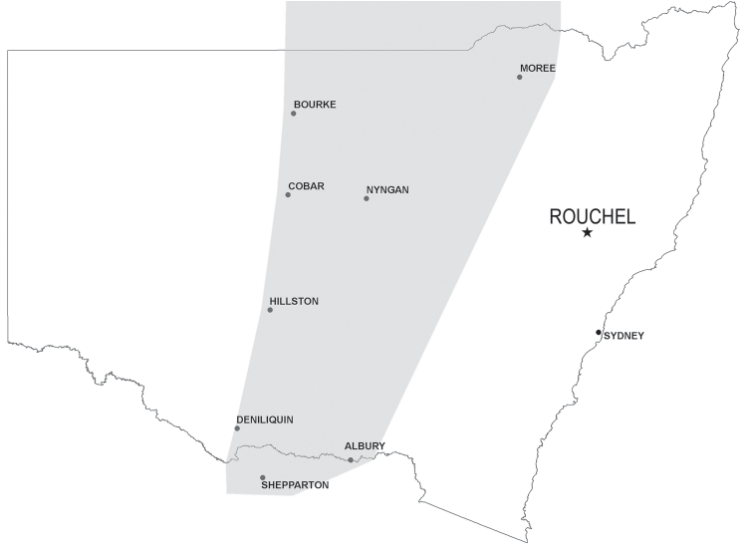
Map of New South Wales, Australia, showing the anthrax belt (gray shading) and the Rouchel location where anthrax reemerged during December 14, 2007–January 3, 2008.

The animals that died in this area were of all ages. Anthrax was not suspected because of a long history of no local activity, but *Bacillus anthracis* was initially confirmed by microscopy of blood smears and subsequently by PCR of blood or other carcass fluid smear scrapings taken when animals were decomposed and microscopy was unreliable (*4*). Dates of discovery of the index case on each property ranged from December 14 to December 29; 1–26 deaths (median 2) occurred per property. Property attack rates varied from 0.9% (1/110 cattle) to 10.7% (3/28 cattle). All stock on infected and 24 neighboring properties were vaccinated in late December; carcasses were burned to ash; movement control, including quarantine, was implemented; and all subsequent stock deaths in the area were investigated to rule out anthrax. One subsequent case occurred when an unvaccinated bull was introduced onto an infected property in late May 2008.

Detailed record review excluded importation of infected feed from known anthrax-endemic areas before the outbreak, and no deaths occurred in stock introduced from these areas during the previous month. Many of the animals died near streams, and waterborne spore dispersal with infection was initially hypothesized. However, the temporal pattern of properties affected, with downstream properties affected before upstream properties; the fact that properties without contiguous streams were affected; and the dilution effect of rapidly flowing streams argued against this transmission route. Because they are septicemic, terminally ill animals with anthrax often seek water (*5*).

The mysterious contemporaneous reemergence of anthrax in this area is unlikely to be explained by mechanical vector-borne transmission because only 1 animal had eye damage, suggesting a crow attack. There was no additional evidence of scavenger attack. No tabanid species (biting) flies were seen on any carcass, and the small number of carcasses and relatively large distances between some properties made mechanical transmission with ocular inoculation by nonbiting flies unlikely (*4*).

Both the remarkable survival capability of anthrax spores and a 1-in-100-year rain event probably contributed to the near-simultaneous reemergence of anthrax on multiple properties in this area. Anthrax spores are resistant to biological extremes of heat, cold, pH, desiccation, chemicals, and irradiation, persisting in this state for decades awaiting conditions that favor germination and multiplication (*6*). In June 2007, drought-ravaged Hunter Valley experienced intense flooding; most rain fell in just 3 days (259 mm in the Aberdeen area, compared with the previous 3-year June average of 43 mm), and massive amounts of topsoil washed into gullies and streams. During late 2007, rainfall also was excessive: 132 mm and 129 mm in November and December, respectively, compared with the 3-year average of 87 mm and 65 mm.

The June floods are likely to have unearthed anthrax spores in the area. The question remains whether these spores had been present for >6 decades, concentrating in depressions that collected water and dead vegetation, potentially providing a milieu for germination and multiplication (i.e., incubator areas), a mechanism that has been implicated in wildlife epidemics of anthrax (*7**,**8*). Alternatively, low-grade sporadic infection may have been ongoing since the 1940s and infrequent stock mortality may not have been investigated for anthrax because of a low local index of suspicion, resulting in environmental contamination The extreme weather conditions in the area may have unearthed spores from undiagnosed carcasses, providing simultaneous exposures on multiple properties.

**We are currently unable to resolve this epidemiologic conundrum. However, our experience is a timely reminder that** veterinary public health authorities should be on high alert for possible anthrax when unexpected livestock deaths follow flooding in areas where anthrax has historically occurred.
